# Trombose Tardia de Prótese Biológica em Posição Mitral com Apresentação com Obstrução Significativa e Insuficiência Cardíaca Aguda

**DOI:** 10.36660/abc.20220481

**Published:** 2023-03-20

**Authors:** Ana Filipa Cardoso, Tamara Pereira, Filipa Cordeiro, Marina Fernandes, Olga Azevedo, António Lourenço

**Affiliations:** 1 Departamento de Cardiologia Hospital Senhora da Oliveira Guimarães Portugal Departamento de Cardiologia, Hospital Senhora da Oliveira, Guimarães – Portugal

**Keywords:** Valva Mitral/cirurgia, Trombose/complicações, Próteses Valvulares Cardíaca/cirurgia, Ecocardiografia/métodos, Insuficiência Cardíaca, Anticoagulantes

## Introdução

A trombose de prótese valvar biológica (TPVB) tem uma incidência que varia entre 0,5% e 6%. No entanto, ela provavelmente é subnotificada devido às limitações técnicas da ecocardiografia transtorácica (ETT) 2D e ao desconhecimento da condição.^1^

Relatamos o caso de um homem de meia-idade que foi internado com insuficiência cardíaca aguda e foi diagnosticado com TPVB. O reconhecimento do quadro e a adoção imediata da anticoagulação parenteral permitiram o restabelecimento do funcionamento normal da válvula biológica, evitando estratégias invasivas.

## Relato de caso

Um homem de 52 anos deu entrada no hospital com insuficiência cardíaca aguda. Três anos antes, ele foi submetido a cirurgia de substituição da válvula mitral por uma prótese biológica de 31 mm (STM Epic™), devido a endocardite de valva nativa. Seu ETT 6 meses antes mostrava uma prótese não obstruída com um gradiente médio de pressão transvalvar (GMPT) de 5 mmHg. Seu histórico médico também incluía fibrilação atrial (FA) paroxística, com um único episódio documentado no período pós-operatório. A anticoagulação com antagonista da vitamina K (AVK) foi interrompida 18 meses após a cirurgia devido a um escore CHA_2_DS_2_-VASc de 0. Ele estava assintomático até a internação atual, quando apresentou dispneia de início agudo. Ele negou a ocorrência de dor torácica ou febre.

À admissão apresentava pressão arterial de 100/65 mmHg, frequência cardíaca de 90 batimentos/min em ritmo sinusal e saturação periférica de oxigênio de 95% a 5 l/min por cânula nasal. Ele estava apirético. Apresentava dificuldade respiratória e estertores dispersos à ausculta pulmonar. O exame cardíaco não revelou anormalidades além de sons cardíacos abafados. Não havia outras anormalidades ao exame físico.

**Figura 1:**
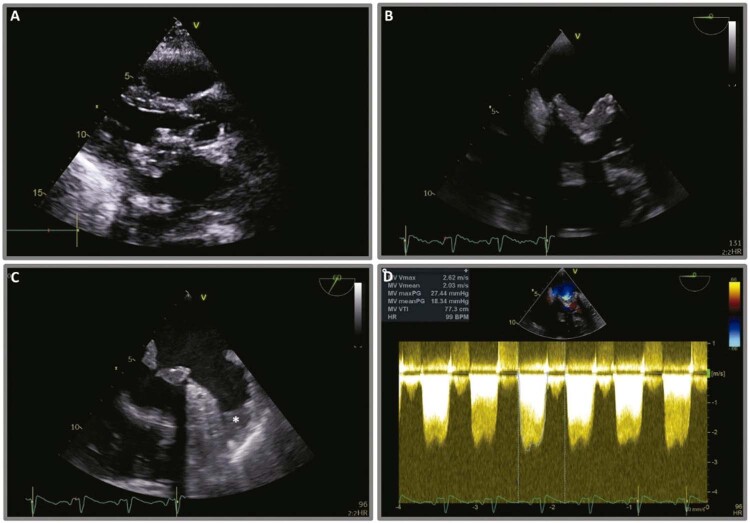
– (A) Vista paraesternal eixo longo de ecocardiografia transtorácica basal. (B) Vista ampliada de ecocardiografia transesofágica da valva mitral em 0º e (C) em 60º, confirmando um espessamento anormal dos folhetos (*denota um trombo no apêndice atrial esquerdo). (D) Doppler de onda contínua revelando gradiente médio de pressão transvalvar de 18 mmHg.

Os exames de sangue revelaram um nível sérico de lactato de 4 mmol/L, contagem normal de leucócitos e proteína C-reativa elevada (14 mg/L, normal <3 mg/L). O eletrocardiograma estava normal. O ETT mostrou folhetos de protése biológica valvar significativamente espessados e um GMPT de 19 mmHg ( Figura 1A ). Não se observou regurgitação valvular, vegetações ou alterações significativas nas outras válvulas. A fração de ejeção ventricular esquerda estava preservada.

O paciente iniciou ventilação não invasiva e diuréticos intravenosos, e foi estabilizado. Foi iniciado o tratamento com heparina não fracionada e antibióticos empíricos para endocardite protética tardia, e ele foi internado.

A ecocardiografia transesofágica (ETE) revelou espessamento acentuado dos folhetos e aparência heterogênea do aspecto ventricular da válvula, compatível com trombose de prótese. Havia um trombo visível no apêndice atrial esquerdo ( Figura 1B-D ; Vídeo 1 ). Não foram observadas vegetações.

**Figura 2:**
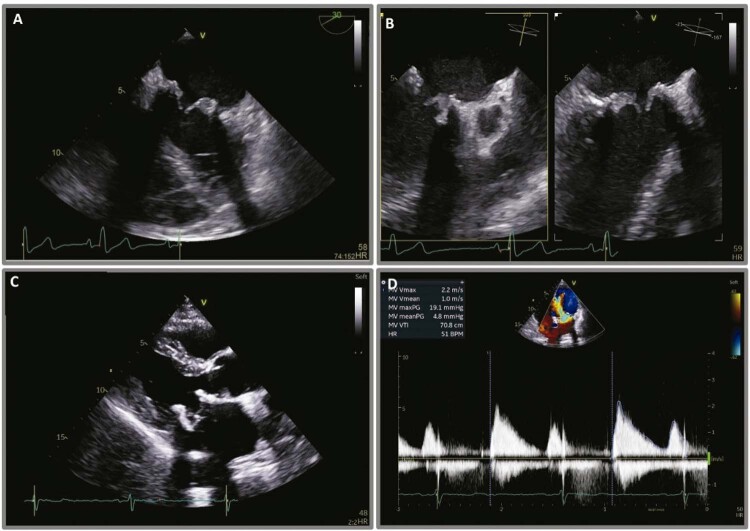
– (A) e (B) - Vistas diferentes de ecocardiografia transesofágica realizada no 10º dia de anticoagulação mostrando folhetos mais finos. (C) Ecocardiografia transtorácica realizada 15 dias após a alta, mostrando espessura normal da prótese valvar biológica. (D) Doppler de onda contínua documentando um gradiente médio de pressão transvalvar de 5 mmHg.

Os antibióticos foram suspensos após hemoculturas seriadas negativas, e o paciente foi mantido em anticoagulação sob rigorosa vigilância ecocardiográfica. Ele não apresentou sintomas de insuficiência cardíaca a partir do 6º dia de internação hospitalar. Uma reavaliação do ETE no 10º dia mostrou redução significativa da espessura dos folhetos ( Figura 2A-B ).

**Vídeo 1:**
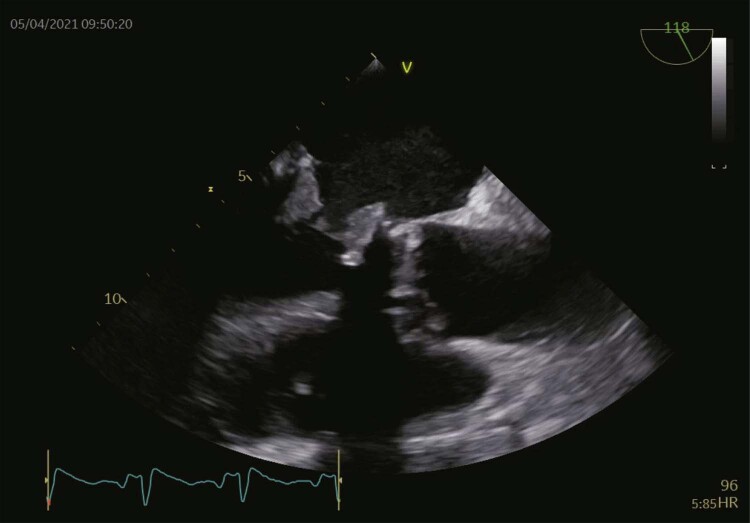
– Vista da ecocardiografia transesofágica a 180° mostrando uma aparência heterogênea do aspecto ventricular da válvula, com espessamento acentuado anormal dos folhetos.

Link: http://abccardiol.org/supplementary-material/2023/12003/2022-0481_CC_Video1.mp4

Após 17 dias de anticoagulação parenteral, o GMPT se normalizou. O paciente passou a tomar a anticoagulação oral com varfarina e teve alta.

Uma ETT de reavaliação indicou a manutenção de folhetos finos, um GMPT de 5 mmHg e ausência de outras complicações locais ( Figura 2C-D ).

O exame de hipercoagulabilidade do paciente deu negativo. Ele se manteve assintomático e em tratamento com varfarina durante o acompanhamento.

## Discussão

Este relato demonstra um caso de TPVB mitral obstrutiva de início tardio tratado com sucesso com anticoagulação parenteral.

Os mecanismos para TPVB não são completamente compreendidos, mas envolvem perturbações do fluxo sanguíneo, resultando em alta tensão de cisalhamento viscoso, ativação de fatores hemostáticos e fatores relacionados ao paciente.^1^O impacto de diferentes fatores para a fisiopatologia da TPVB provavelmente varia com a localização da prótese, refletindo distintas anatomias valvares e condições de fluxo. Para a válvula mitral, fatores hemodinâmicos, particularmente fluxo sanguíneo lento em pacientes com FA ou baixo débito ventricular esquerdo, parecem ser importantes, mas são necessárias mais pesquisas sobre os possíveis mecanismos.^2^

Há falta de uma definição universal para a TPVB. Egbe et al.,^3^ propuseram um modelo que incluía variáveis clínicas (incluindo a presença de FA paroxística e uma razão normalizada internacional subterapêutica) e parâmetros ecocardiográficos (aumento de 50% no GMPT no período de 5 anos após cirurgia; aumento da espessura da cúspide e mobilidade da cúspide alterada).^3^A distinção ecocardiográfica de TPVB da degeneração estrutural pode ser difícil. Geralmente, o trombo se apresenta como um material macio e denso no lado ventricular das válvulas atrioventriculares ou no lado arterial das válvulas semilunares, enquanto as válvulas degeneradas apresentam espessamento de cúspide mais ecodenso, mas menos proeminente.^3^ A tomografia computadorizada cardíaca (TCC) é útil para o diagnóstico diferencial com crescimento do pannus, uma causa de disfunção da prótese que se apresenta com valores de atenuação da TCC mais elevados.^4 , 5^ Sua alta precisão também permite a detecção de TPVB subclínica, identificando o espessamento hipoatenuante dos folhetos, uma condição reconhecida principalmente após o implante de válvula aórtica transcateter, mas também relatada após a implantação cirúrgica de válvula mitral biológica.^6^

O tratamento da TPVB depende da apresentação clínica, da repercussão hemodinâmica e da válvula acometida. Recomenda-se a anticoagulação com AVK ou heparina não fracionada antes de considerar uma nova intervenção.^4^

Existe a percepção de que a TPVB é um fenômeno perioperatório cujo risco é aumentado até a endotelização do material protético. As diretrizes da European Society of Cardiology/European Association for Cardio-Thoracic Surgery (ESC/EACTS) e do American College of Cardiology/American Heart Association (ACC/AHA) recomendam a anticoagulação com AVK por 3 a 6 meses após a implantação cirúrgica de uma válvula mitral biológica em pacientes com baixo risco de sangramento.^4 , 7^ No entanto, algumas séries relataram uma incidência mediana de pico da TPVB aos 34 meses após o implante.^8^ Nosso caso corrobora a manutenção da suspeita diagnóstica em um cenário clínico apropriado, independentemente do tempo desde a implantação.

As recomendações para terapia antitrombótica de longo prazo após a implantação cirúrgica da válvula mitral biológica variam. Enquanto as diretrizes do ACC/AHA apoiam a administração de aspirina em baixas doses durante toda a vida, as diretrizes da ESC/EACTS só a recomendam se houver indicação de terapia antiplaquetária concomitante.^4 , 7^ Não há recomendações específicas para pacientes com FA com baixo risco tromboembólico. Esse foi o caso de nosso paciente, que apresentou uma carga trombótica significativa, apesar de ter um escore CHA_2_DS_2_-VASc de 0. Novos estudos abordando essa população específica seriam importantes para esclarecer o manejo mais adequado.

As diretrizes atuais recomendam imagens de rotina após 5 anos (ACC/AHA) ou anualmente (ESC/EACTS) após o implante cirúrgico de válvulas biológicas.^4 , 7^ Como a TPVB pode ocorrer nos primeiros anos após o implante e em pacientes minimamente sintomáticos, uma estratégia de vigilância menos conservadora provavelmente deixará passar alguns diagnósticos.

Especula-se que a TPVB preceda a degeneração valvular precoce, mesmo após terapia bem-sucedida. Um estudo anterior mostrou um risco 3,2 vezes maior de ser necessária uma nova cirurgia de substituição da válvula durante o acompanhamento de longo prazo. A recorrência é descrita em até 23% dos casos, confirmando a indicação de anticoagulação vitalícia.^4 , 9^ Embora não exista nenhuma recomendação específica de acompanhamento para esses pacientes, uma vigilância mais rigorosa pode ser apropriada.^7^

## Conclusão

As próteses valvares biológicas têm um baixo risco percebido de trombose, fazendo com que o diagnóstico da TPVB seja um desafio e que a TPVB seja uma causa pouco reconhecida de insuficiência valvular aguda ou indolente.^1 , 2^Essa condição pode ocorrer anos após a cirurgia e deve ser incluída no diagnóstico diferencial de pacientes que apresentam aumento do GMPT, insuficiência cardíaca ou eventos tromboembólicos.^1 , 2 , 4^Conforme relatado neste caso, a anticoagulação pode reverter a disfunção valvar, fazendo com que seja importante considerar a TPVB antes de encaminhar os pacientes para uma nova intervenção.
