# Supplementary material for: Trombose Tardia de Prótese Biológica em Posição Mitral com Apresentação com Obstrução Significativa e Insuficiência Cardíaca Aguda
Source: Arq Bras Cardiol. 2023 Mar 20;120(3):e20220481. [Article in Portuguese] doi: 10.36660/abc.20220481 (PMC10392860; doi:10.36660/abc.20220481)
Supplement: Translation

# Translation: Late-Onset Bioprosthetic Mitral Valve Thrombosis, Presenting with Significant Obstruction and Acute Heart Failure

**Authors:** Ana Filipa Cardoso, Tamara Pereira, Filipa Cordeiro, Marina Fernandes, Olga Azevedo, António Lourenço

**Affiliations:** 1 Portugal Departamento de Cardiologia, Hospital Senhora da Oliveira, Guimarães – Portugal

**Keywords:** Mitral Valve/surgery, Thrombosis/complications, Heart Valve Prosthesis/surgery, Echocardiography/methods, Heart Failure, Anticoagulants

## Introduction

Bioprosthetic valve thrombosis (BPVT) has an incidence ranging from 0.5% to 6%. However, it is most likely underreported given the technical limitations of 2D transthoracic echocardiography (TTE) and the unawareness of the condition.^1^

Our study presents a case report of a middle-aged male who was admitted with acute heart failure and was diagnosed with BPVT. Recognition of the condition and prompt institution of parenteral anticoagulation allowed for the restoration of the normal functioning of the biological valve, avoiding invasive strategies.

## Case report

A 52-year-old male was admitted to the hospital with acute heart failure. Three years before, he had undergone a mitral valve replacement surgery using a 31mm biological prosthesis (STM Epic^™^) due to native valve endocarditis. His TTE 6 months earlier had shown a non-obstructed prosthesis with a transvalvular mean pressure gradient (TMPG) of 5mmHg. His medical history also included paroxysmal atrial fibrillation (AF), with a single documented episode in the postoperative period. Anticoagulation with vitamin K antagonist (VKA) was stopped 18 months after surgery due to a CHA_2_DS_2_-VASc score of 0. He was asymptomatic until the current admission, when he presented an acute onset of dyspnoea. He denied feeling any chest pain or fever.

Upon admission, his blood pressure was 100/65 mmHg, heart rate 90 beats/min in sinus rhythm, and peripheral oxygen saturation 95% on 5l/min by nasal cannula. He was apyretic, was in respiratory distress, and presented dispersed crackles upon pulmonary auscultation. Cardiac examination revealed no abnormalities besides muffled cardiac sounds. No other abnormalities were found upon physical examination.

Blood tests revealed a serum lactate level of 4 mmol/L, a normal white blood cell count, and high levels of C-reactive protein (14mg/L, normal<3 mg/L). The electrocardiogram was unremarkable. TTE showed significantly thickened bioprosthetic valve leaflets and a TMPG of 19mmHg (Figure 1A). No valvular regurgitation, vegetations or significant abnormalities were found on the other valves. Left ventricular ejection fraction was preserved.

The patient initiated non-invasive ventilation and intravenous diuretics, and was stabilized. Unfractionated heparin and empirical antibiotics for late prosthetic endocarditis were started, and he was admitted to the hospital.

Transoesophageal echocardiography (TEE) showed a marked thickening of the leaflets and a heterogeneous appearance of the ventricular aspect of the valve, compatible with prosthesis thrombosis. A thrombus was visible on the left atrial appendage ( Figure 1B-D Video 1 ). No vegetations were noted.

**Figure 1:** – (A) Baseline transthoracic echocardiography parasternal long-axis view. (B) Transoesophageal echocardiography zoom view of mitral valve at 0º and (C) at 60º, confirming abnormal thickening of the leaflets (*denotes a left atrial appendage thrombus). (D) Continuous wave Doppler revealing a transvalvular mean pressure gradient of 18mmHg.

**Video 1:** – Transoesophageal echocardiography view at 180º showing a heterogenous appearance of the ventricular aspect of the mitral valve with marked abnormal thickening of the leaflets.

Link: Link: http://abccardiol.org/supplementary-material/2023/12003/2022-0481_CC_Video1.mp4

Antibiotics were stopped after serial negative blood cultures, and the patient was kept on anticoagulation under close echocardiographic surveillance. He remained free from heart failure symptoms from the 6^th^day of hospitalization on. A re-evaluation TEE on the 10^th^day showed a significant reduction in the leaflet thickness ( Figure 2A-B ).

**Figure 2:** – (A) and (B) - Different views of transoesophageal echocardiography performed on the 10th day of anticoagulation, showing thinner leaflets. (C) Transthoracic echocardiography performed 15 days after discharge, showing normal thickness of the bioprosthetic valve. (D) Continuous wave Doppler, documenting a transvalvular mean pressure gradient of 5mmHg.

After 17 days of parenteral anticoagulation, the TMPG had normalized. The patient was switched to oral anticoagulation with warfarin and discharged.

A re-evaluation TTE maintained thin leaflets, a TMPG of 5mmHg, and no other local complications ( Figure 2C-D ).

The patient’s hypercoagulable workup came back negative. He remained asymptomatic and under warfarin treatment during the follow-up.

## Discussion

This report demonstrates a case of late-onset obstructive mitral BPVT successfully treated with parenteral anticoagulation.

The mechanisms for BPVT are not completely understood, but they involve blood flow perturbations resulting in high viscous shear stress, activation of hemostatic factors and patient-related factors.^1^The impact of different factors for BPVT pathophysiology likely varies with prosthesis location, reflecting distinct valve anatomies and flow conditions. For the mitral valve, hemodynamic factors, particularly slow blood flow in patients with AF or low left ventricular output, seem to be important, but further research into potential mechanisms is needed.^2^

There is no clear universal definition for BPVT. Egbe et al.^3^proposed a model that included clinical variables (including the presence of paroxysmal AF and a subtherapeutic international normalized ratio) and echocardiographic parameters (50% increase in the TMPG within 5-years of surgery; increased cusp thickness, and abnormal cusp mobility).^3^Echocardiographic distinction of BPVT from structural degeneration may be difficult. Usually, the thrombus appears as a soft dense material on the ventricular side of atrioventricular valves or arterial side of semilunar valves, whereas degenerated valves appear with more echodense but less prominent cusp thickening.^3^Cardiac computed tomography (CCT) is useful for differential diagnosis with pannus ingrowth, a cause of prosthesis dysfunction that appears with higher CCT attenuation values.^4 , 5^Its high accuracy also allows for the detection of subclinical BPVT by identifying hypoattenuated leaflet thickening, a condition mainly recognized after transcatheter aortic valve implantation, but also reported after a surgically-implanted biological mitral valve.^6^

The treatment of BPVT depends on the clinical presentation, hemodynamic impact, and valve affected. Anticoagulation with VKA or unfractionated heparin is recommended before considering re-intervention.^4^

There is a perception that BPVT is a perioperative phenomenon whose risk is increased until prosthetic material endothelization. The European Society of Cardiology/European Association for Cardio-Thoracic Surgery (ESC/EACTS) and the American College of Cardiology/American Heart Association (ACC/AHA) guidelines recommend anticoagulation with VKA for 3 to 6 months after a surgically-implanted biological mitral valve in low-bleeding risk patients.^4 , 7^Nevertheless, some series have reported a median peak incidence of BPVT at 34 months after implantation.^8^Our case supports maintaining diagnostic suspicion in an appropriate clinical scenario, regardless of the time since implantation.

Recommendations for long-term antithrombotic therapy after surgically-implanted biological mitral valve differ. While ACC/AHA guidelines support lifelong low-dose aspirin administration, ESC/EACTS guidelines only recommend it if there are concomitant indications for antiplatelet therapy.^4 , 7^No specific recommendations are given for AF patients with low thromboembolic risk. This was the case with our patient, who as admitted with a significant thrombotic load despite a CHA_2_DS_2_-VASc score of 0. Further studies addressing this specific population would be important to clarify the most appropriate management.

Current guidelines recommend routine imaging after 5 years (ACC/AHA) or yearly (ESC/EACTS) after surgically-implanted biological valves.^4 , 7^Since BPVT can occur in the early years after implantation and in minimally-symptomatic patients, a less conservative surveillance strategy will probably miss some diagnoses.

BPVT is speculated to precede early valve degeneration, even after successful therapy. A previous study showed a 3.2-fold increased risk of valve re-replacement surgery during long-term follow-up. Recurrence is described in up to 23% of the cases, supporting lifelong anticoagulation.^4 , 9^Although no specific follow-up recommendation exists for these patients, closer surveillance may be appropriate.^7^

## Conclusion

Bioprosthetic valves have a low perceived risk of thrombosis, making BPVT a challenging diagnosis and an under-recognized cause of acute or indolent valve failure.^1 , 2^This condition can occur years after surgery and should be included in the differential diagnosis of patients who present a rising TMPG, heart failure, or thromboembolic events.^1 , 2 , 4^As reported in this case, anticoagulation may reverse valve dysfunction, making it important to consider BPVT before referring patients for re-intervention.
